# Thoracic and Abdominal Radiologic Anatomy of the Greater Cane Rat (*Thryonomys swinderianus*)

**DOI:** 10.1155/vmi/5558928

**Published:** 2026-07-14

**Authors:** Mazengo Masigati, Mirende Kichuki, Modesta Makungu

**Affiliations:** ^1^ Department of Veterinary Surgery and Theriogenology, College of Veterinary Medicine and Biomedical Sciences, Sokoine University of Agriculture, P.O. Box 3020, Morogoro, Tanzania, suanet.ac.tz

**Keywords:** abdomen, anatomy, diagnostic imaging, radiography, rodentia, thorax

## Abstract

The greater cane rat (*Thryonomys swinderianus*) is widely distributed in sub‐Saharan Africa, including western, central, eastern and southern Africa. It is the African’s second‐largest rodent. This study was done to describe and establish the normal reference values for radiographic anatomy of the thorax and abdomen in greater cane rats for biomedical and related research, teaching comparative veterinary anatomy and clinical uses. Radiography of the thorax and abdomen was performed under general anaesthesia in ten (10) wild adult greater cane rats. Right lateral (RL), left lateral (LL), dorsoventral (DV) and ventrodorsal (VD) radiographic projections of the thorax were taken. For the abdomen, RL and VD projections were obtained. All greater cane rats had 13 thoracic vertebrae with corresponding pairs of ribs. The number of lumbar vertebrae was mainly six. The sternum consisted of manubrium sterni, xiphoid process and four sternebrae. The anticlinal vertebra was mainly the 11th thoracic vertebra, and the sacrum consisted of four fused sacral vertebrae. Reference ranges for thoracic and abdominal organs/structures were established, such as the tracheal diameter (6.8 mm), angle between the mainstem bronchi (DV: 47.8°; VD: 46.4°), and gastric axis angle (77.6°). Further, ratios like the vertebral heart size (VHS) (RL: 9.70, LL: 9.83, DV: 10.46 and VD: 10.45), caudal vena cava diameter to length of thoracic vertebra above the carina (0.86), tracheal diameter to thoracic inlet diameter (0.32), and cardiothoracic (DV: 0.63, VD: 0.64) were calculated. Measurements of sternebrae, thoracic and lumbar vertebrae are provided. Differences and similarities with other rodents have been highlighted. Information from this research will serve as a reference for routine veterinary clinical procedures and biomedical studies and for teaching veterinary comparative anatomy and diagnostic imaging. Additionally, the use of ratios of absolute measurements of thoracic and abdominal organs/structures to relevant skeletal structures is recommended to overcome intraspecies variability in body size.

## 1. Introduction

The greater cane rat (*Thryonomys swinderianus*) is Africa’s second‐largest rodent after the porcupine (*Hystrix africaeaustralis*) [1]. It belongs to the order Rodentia, family Thryonomidae and genus *Thryonomys* [2]. Greater cane rats are widely distributed in sub‐Saharan Africa, including western, central, eastern and southern Africa [2–4]. The cultural practice of bushmeat consumption, the decline in harvested wildlife species, and increased bushmeat demand have led to increased efforts in the domestication of greater cane rats [5–7]. Further, their use in biomedical research increases in their domestication demand [8, 9].

Knowledge paucity on proper management practices, reproductive physiology and diseases hinders domestication efforts of the greater cane rat [1]. Thoracic and abdominal diseases such as pneumonia, gastrointestinal obstruction, gastroenteritis, hepatitis, neoplasia and aflatoxicosis have been reported in captive‐reared greater cane rats [10–13]. Additionally, rats have been reported to be prone to heart diseases [14–17].

Diagnostic radiography is a non‐invasive imaging modality that has been in use in both human and veterinary practices for the diagnosis, treatment and monitoring of the treatment response of different diseases and conditions [18, 19]. Diseases and conditions affecting the musculoskeletal system and other body systems such as the cardiovascular, digestive, respiratory and lymphatic systems, have been reported to be diagnosed by radiography [18, 19]. The sound knowledge of the normal radiographic anatomy of the thorax and abdomen of a specific animal species enhances the effective use of diagnostic radiography as a diagnostic tool [15, 18, 20–23].

The majority of studies on radiographic anatomy of the thorax in rodents have been based on determination of the vertebral heart size (VHS) and cardiothoracic ratios [15, 24–27]. Additionally, there are a few reports that have documented a comprehensive normal radiologic anatomy of the thorax and abdomen in rabbits and rats [23, 28–30]. These studies have highly contributed to the use of radiography in detection of thoracic diseases and conditions like vertebral degenerative changes, respiratory infections, pneumothorax, pulmonary oedema, pleural effusion, cardiomegaly, lung metastases, and pulmonary bulla in respective species [31–35]. In addition, radiography has been reported to diagnose abdominal diseases and conditions such as lower urinary tract diseases, small intestinal obstruction, neoplasia and gastric dilatation and volvulus in domestic rats, guinea pigs and rabbits [36–39]. A previous radiological study on the greater cane rat thorax [40] was based on cardiac mensuration. Recent publications on radiological studies in the greater cane rat have been based mainly on the identification of anatomical structures/organs and barium transit through the gastrointestinal tract [41, 42]. Nevertheless, to the best of the authors’ knowledge, a comprehensive radiological description of the greater cane rat’s thorax and abdomen has not yet been documented.

Therefore, the objectives of this study were to describe the normal thoracic and abdominal radiographic appearance and to establish the normal reference values for thoracic and abdominal organs/structures in greater cane rats. It was hypothesised that significant differences exist in the normal thoracic and abdominal radiological anatomy in rodents. Additionally, understanding the normal thoracic and abdominal radiological anatomy of the greater cane rat will enhance teaching of comparative veterinary anatomy and biomedical and related research. Furthermore, this knowledge leads to contributing to accurate and timely disease diagnosis in this species.

## 2. Materials and Methods

### 2.1. Location of the Study

This study was conducted in the Department of Veterinary Surgery and Theriogenology of the Sokoine University of Agriculture (SUA), College of Veterinary Medicine and Biomedical Sciences (CVMBS). The study was approved by the Research, Innovations, and Publication Committee of the CVMBS and the Directorate of Postgraduate Studies, Research, Technology Transfer and Consultancy (DPRTC) Ethical Committee (DPRTC/R/186/30).

### 2.2. Animals

Ten (10) intact, adult greater cane rats (*T. swinderianus*) with a mean body weight of 3.70 ± 1.73 kg (range: 1.50–7.00 kg) were included in this study. Physical examination and haematologic evaluation were used to assess the health status of cane rats. Of the ten (10) cane rats, four (4) were females and six (6) were males. Female cane rats weighed significantly less than male cane rats (2.13 ± 0.60 kg vs. 4.75 ± 1.37 kg, *p* = 0.0074). The animals were captured from their natural habitat using traps and transported to the Department of Veterinary Surgery and Theriogenology, SUA. They were kept at the animal house for 48 h before radiographic examination for acclimatisation. During this period, water and food were given *ad libitum*, with their behaviour monitored.

### 2.3. Radiographic Examinations

Radiographic examinations of the thorax were performed immediately after administration of general anaesthesia, followed by abdominal radiography. In each cane rat, xylazine (Interchemie Werken, Holland) as a sedative in combination with ketamine hydrochloride (Troikaa Pharmaceuticals Ltd., India) as a general anaesthetic was used [43]. The drugs were administered intramuscularly at doses of 0.5 mg/kg body weight (xylazine) and 20 mg/kg body weight (ketamine hydrochloride). Tracheal intubation was not performed, and cane rats were under natural spontaneous respiration throughout the radiographic examination. The vital signs for anaesthetised cane rats were closely monitored until full recovery. The xylazine and ketamine combination was selected due to its efficacy in rodents [43].

**FIGURE 1:**
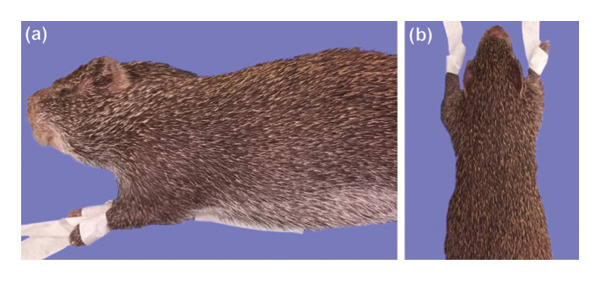
Photographs of an adult 2.5 kg greater cane rat in a right lateral (a) and sternal (b) recumbencies illustrating positioning for a right‐lateral (RL) and dorsoventral (DV) thoracic radiographic projections, respectively.

Four thoracic radiographic projections, right lateral (RL), left lateral (LL), dorsoventral (DV) and ventrodorsal (VD), were obtained during inspiration by manual inflation of the lungs. Inflation was achieved using a face mask connected to a self‐inflating bag, ensuring visible thoracic expansion without causing hyperinflation. Collimation included the thoracic inlet cranially and extended caudally to contain the cranial abdomen. For the LL and RL radiographic projections, cane rats were positioned in LL and RL recumbencies, respectively (Figure 1a), and the X‐ray beam was centred just caudal to the scapula [23]. The thoracic limbs and pelvic limbs were extended cranially and caudally, respectively, with the thoracic limbs secured using masking tape to avoid superimposition of the elbow joint muscles on the cranial thoracic structures (Figure 1a). A mean kilovoltage peak (kVp) of 62.50 ± 4.84 (range: 50–66 kVp) and 2 milliampere‐seconds (mAs) were used. For VD and DV radiographic projections, greater cane rats were positioned in dorsal and sternal recumbencies, respectively (Figure 1b). The pelvic limbs were flexed, whereas the thoracic limbs were pulled cranially and secured with masking tape (Figure 1b). The X‐ray beam was centred on the centre of the sternum for the VD projection. For the DV radiographic projection, the X‐ray beam was centred between the scapulae and just at their caudal aspect [23]. A mean kVp of 63.90 ± 4.70 (range: 52–68 kVp) and 2 mAs were applied for the VD and DV radiographic projections.

For the abdomen, RL and VD radiographic projections were acquired at the end of the expiratory phase. Cane rats were positioned in dorsal and RL recumbencies for the VD and RL radiographic projections, respectively [44]. The pelvic limbs were flexed in a frog‐legged position for the VD projection, whereas for the RL projection they were pulled caudally [44]. Collimation included the caudal thorax cranially and extended to the greater trochanters caudally. The x‐ray beam was centred just caudal to the last rib [44]. A mean kVp of 47.00 ± 1.25 (range: 46–50 kVp) and 4 mAs were applied.

All radiographs were obtained at a source‐to‐image distance (SID) of 100 cm using a Roller 30 X‐ray machine (Smam X‐ray Equipments, Italy), and a nongrid technique was applied. Standard Fuji imaging plates (CRST‐VI, Fujifilm Corporation) sizes 24 cm × 30 cm and 35.4 cm × 43.0 cm were used depending on the size of the greater cane rat. Acquired images were processed using the ColentaHighCapXr^®^ (Fuji Film Corporation, Japan) computed radiography (CR) system (greyscale resolution: 12 bit; reading specification: 10 pixel/mm, 5 pixel/mm; pixel size: 100 μm).

### 2.4. Evaluation of Radiographs

Evaluation of thoracic and abdominal radiographs was performed using Colenta Dx Easy Imaging AQS Version 2.10.31 (COLENTA Labortechnik GmbH & Co KG, Austria) software on an HP Elite Display E221 monitor (Qisda [Suzhou] Co. Ltd, China). Radiographs of good quality, with properly positioned cane rats without evidence of thoracic and/or abdominal disease, were included in the study. The thoracic and abdominal organs/structures were evaluated based on roentgen signs, that is, visibility, location, number, size, shape, margination and opacity [18].

#### 2.4.1. Thorax

##### 2.4.1.1. Shape, Detail and Size

**FIGURE 2:**
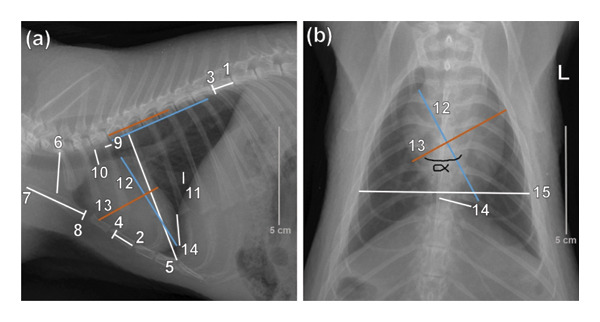
Right‐lateral (a) and dorsoventral (b) thoracic radiographs of a 3.5 kg (a) and 5 kg (b) adult greater cane rat, illustrating radiographic measurements. (1) Length of thoracic vertebral body, (2) length of sternebra, (3) height of thoracic vertebral body, (4) height of sternebra, (5) thoracic depth, (6) thoracic inlet diameter, (7) length of the manubrium sterni, (8) height of the manubrium sterni, (9) third rib width, (10) tracheal diameter, (11) caudal vena cava diameter, (12) cardiac silhouette long‐axis, (13) cardiac silhouette short‐axis, (14) cardiodiaphragmatic contact, (15) thoracic width and (ɑ) angle between the mainstem bronchi. L, left.

The shape of the thorax and visibility of thoracic structures were noted in each greater cane rat on the lateral, VD and DV projections (Figure 2). Additionally, for each cane rat, thoracic width, depth and inlet diameter were measured, and the thoracic depth‐to‐width ratio was calculated. Thoracic width (maximum lateral diameter) was measured between the medial surfaces of the sixth ribs (Figure 2b) [23]. Thoracic depth was measured from the caudodorsal border of the fifth sternebra to the ventral surface of the thoracic vertebra (Figure 2a) [23]. Thoracic inlet diameter was measured on RL projections as the distance between the dorsal midpoint of the manubrium sterni and the ventral surface of the first thoracic vertebra (Figure 2a).

##### 2.4.1.2. Musculoskeletal System

In all cane rats, the shape and number of the sternebrae, ribs and thoracic vertebrae were noted. Additionally, height and length measurements were performed for the sternebrae and thoracic vertebrae. The anticlinal vertebra, which is the point in the caudal thoracic spine at which vertebral anatomical features change, was identified on the RL projection [23]. The height and length measurements of sternebrae and thoracic vertebrae were made on the RL projection (Figure 2a) [23]. The height was measured on the cranial endplate (Figure 2a), whereas the length was measured from the midpoint of the cranial endplate to the midpoint of the caudal endplate (Figure 2a) [23]. For the manubrium sterni, the maximum length (Figure 2a) was measured from the most cranial endpoint to the midpoint of the caudal endplate and its height (Figure 2a) was determined from the caudal endplate because of the rounded to pointed appearance of its cranial extremity. Similarly, the length of the xiphoid process was measured from the midpoint of the cranial endplate to the most caudal end due to a rounded to pointed appearance of its caudal extremity. Third rib width (Figure 2a) was determined at its proximal one‐third from the RL radiographic projection of the thorax [23, 45].

##### 2.4.1.3. Respiratory System

The appearance of the left and right diaphragmatic crura was noted on the lateral, VD and DV thoracic radiographic projections [23]. Additionally, on the VD and DV thoracic radiographic projections (Figure 2b), the crossing point of the most cranial margin of the diaphragmatic cupula to the thoracic vertebra was recorded [23]. On thoracic radiographic projections (Figure 2), the most cranial extension of lung fields was noted with respect to intercostal spaces and ribs [23, 45]. The most caudal extension of lung fields was recorded on the lateral thoracic radiographic projections (Figure 2a) with respect to thoracic vertebrae [23].

Additionally, the tracheal diameter (Figure 2a) was measured perpendicular to its longitudinal axis between the ventral and dorsal margins of its dorsal and ventral walls, respectively, on the RL projection [23, 45]. The tracheal measurement was recorded at the level of the second intercostal space (Figure 2a) and compared with the third rib width and thoracic inlet diameter (Figure 2a) [23, 45, 46]. The position of the carina was noted on the lateral, VD and DV thoracic radiographic projections (Figure 2) [47]. On the DV and VD projections, the carina was recorded with respect to the thoracic vertebra (Figure 2b), whereas on the lateral projections (Figure 2a), it was noted in relation to the thoracic vertebra and intercostal space [23, 45]. The mainstem bronchi intersection point was used to spot the location of the carina in cases where it was invisible [47]. The angle of bifurcation of the left and right mainstem bronchi was recorded between their caudal borders on the VD and DV projections (Figure 2b) [47, 48].

##### 2.4.1.4. Cardiovascular System

The cardiac silhouette (CS), which includes the heart, pericardial contents, pericardium, and the origins of the main pulmonary artery and aorta, was evaluated on the lateral, DV and VD thoracic projections. The CS long‐axis and short‐axis measurements were performed on the lateral, DV and VD thoracic projections (Figure 2). VHS that compares CS dimensions to the thoracic vertebrae length was calculated from long‐axis and short‐axis measurements of the CS [49, 50]. The long‐axis and short‐axis measurements acquired on the LL and RL radiographic projections were equated with thoracic vertebrae on the LL and RL projections, respectively, beginning with the fourth thoracic vertebra (Figure 2a) [23, 49]. For the VD and DV radiographic projections, long‐axis and short‐axis CS measurements (Figure 2b) were equated with thoracic vertebrae on RL projection (Figure 2a) beginning with the fourth thoracic vertebra [23, 49]. Additionally, the CS short axis together with thoracic width measurements on the VD and DV projections (Figure 2b) were used to determine the cardiothoracic ratio, that is, the CS short‐axis to thoracic‐width ratio on the VD and DV projections, respectively [23]. On lateral thoracic radiographs (Figure 2a) the size of the CS was also compared with intercostal spaces [45, 47].

The caudal vena cava (CVC) maximum diameter not overlying the diaphragm or the caudal margin of the CS was recorded as a perpendicular distance to its long axis from the dorsal wall margin to the ventral wall margin on the RL thoracic projection (Figure 2a) [23]. Additionally, the diameter of the CVC (Figure 2a) was compared with the length of the thoracic vertebra positioned above the carina [23]. Cardiodiaphragmatic contact, that is, the contact between the CS and diaphragm (Figure 2), was recorded on the lateral, VD and DV projections [51].

#### 2.4.2. Abdomen

##### 2.4.2.1. Size and Detail

**FIGURE 3:**
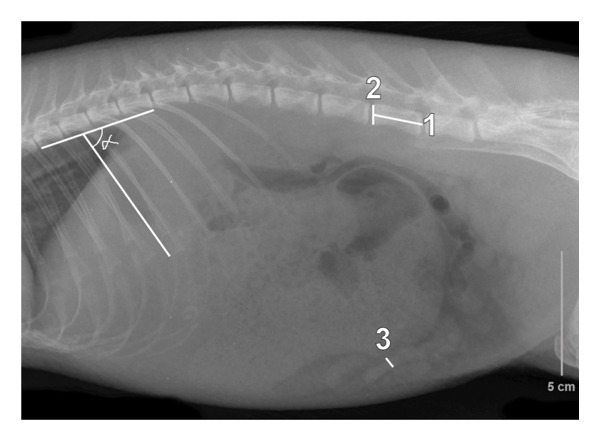
Right‐lateral abdominal radiograph of a 4 kg adult greater cane rat, illustrating radiographic measurements. (1) length of lumbar vertebral body, (2) height of lumbar vertebral body, (3) small intestinal thickness and (ɑ) angle of gastric axis.

The size of the abdominal cavity was evaluated relative to the size of the thoracic cavity. Additionally, the visibility of the abdominal organs was noted on the RL and VD projections (Figure 3).

##### 2.4.2.2. Musculoskeletal System

The shape and number of lumbar and sacral vertebrae were noted on the RL and VD abdominal radiographs (Figure 3). The height and length measurements of lumbar vertebrae (Figure 3) were made on RL abdominal radiographs in a similar manner to that of thoracic vertebrae [23].

##### 2.4.2.3. Digestive System

The gastric axis angle (Figure 3) was recorded on the RL abdominal radiographs as the caudal angle formed between a straight line plotted along the ventral side of thoracic vertebrae 9 to 12 and the gastric axis [20, 21]. The latter is an imaginary line from the fundus through the body and pylorus regions of the stomach [52]. On the RL projection, the small intestinal maximum thicknesses (Figure 3) were measured and compared with the height of the second lumbar vertebra [20, 21].

### 2.5. Data Analysis

Epi Info Version 7.2.6.0 statistical software and Microsoft Office Excel were used for data analysis. Epi Info was used to calculate the range, mean and standard deviation. Additionally, radiographic measurements and weight were compared between male and female greater cane rats. Radiographic measurements in different projections were compared using a paired *t*‐test (Microsoft Office Excel). Statistical significance was established at *p* < 0.05 and data are presented as mean ± standard deviation.

## 3. Results

**FIGURE 4:**
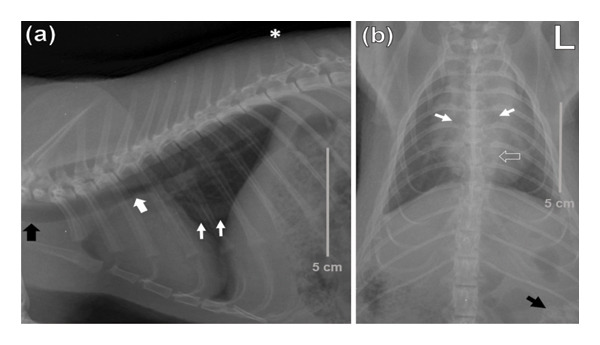
(a) A left‐lateral thoracic radiograph of a 3.5 kg adult greater cane rat. The thoracic cavity is relatively small and almost triangular‐shaped. Note the presence of 13 thoracic vertebrae, six sternebrae and prominent mamillary processes at thoracic vertebrae 12 and 13. Mild mineralisation of the cartilage rings is seen at the caudal cervical trachea (black arrow), and the poorly visualised carina is situated at the third intercostal space (thick white arrow). The ovoid cardiac silhouette is in contact with the diaphragm. The anticlinal vertebra and caudal vena cava are indicated by a white asterisk and thin white arrows, respectively. (b) Ventrodorsal thoracic radiograph of a 5.5 kg adult greater cane rat. The cardiac silhouette is ovoid‐shaped with the apex situated slightly to the left haemithorax. The lateral margin of the descending aorta is indicated by an open white arrow. The left and right mainstem bronchi are indicated by solid white arrows. Grainy materials with mineral opacity are indicated by a black arrow. L, left.

**FIGURE 5:**
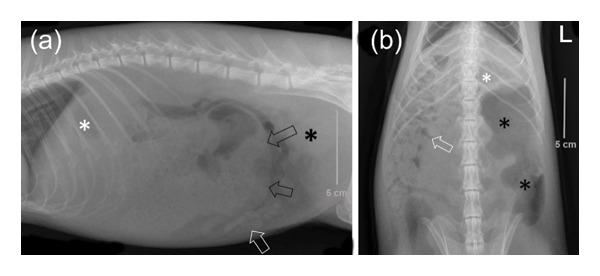
(a) Right‐lateral abdominal radiograph of a 4 kg adult greater cane rat. Note the presence of six lumbar vertebrae with cranially directed spinous processes and prominent mamillary processes superimposed on spinous processes. The abdominal detail is relatively poor, with a prominent caecum (open black arrows). Small intestinal loops are seen in the ventral abdomen (open white arrow). The stomach and an ovoid‐shaped urinary bladder are indicated by white and black asterisks, respectively. (b) Ventrodorsal abdominal radiograph of a 5 kg adult greater cane rat. Note the presence of six lumbar vertebrae. The gas‐filled caecum (black asterisks) occupies a greater proportion of the left abdomen. Small intestinal loops are seen in the right cranial abdomen (white open arrow). The stomach is indicated by a white asterisk. L, left.

Generally, in all greater cane rats, the detail of the thorax was good; the majority of clinically significant structures were seen (Figure 4). Contrarily, most abdominal structures were not clearly seen in all cane rats as a result of poor abdominal detail (Figure 5). The thoracic cavity was more or less triangular (Figure 4) and relatively smaller with abdominal dominance (Figure 5). Thoracic width measured on the DV projection differed significantly from that measured on the VD projection (DV: 70.9 ± 13.88 mm; VD: 68.0 ± 13.81 mm; *p* < 0.0001). Similarly, the ratio of the thoracic depth‐to‐width obtained on the DV projection was also significantly different from the ratio of the thoracic depth‐to‐width obtained on the VD projection (DV: 0.90 ± 0.04; VD: 0.94 ± 0.05; *p* = 0.0001). Gender‐wise, male greater cane rats had a significantly higher mean thoracic depth (72.0 ± 8.10 mm vs. 52.0 ± 9.49 mm, *p* = 0.0072) and width (DV: 79.5 ± 8.24 mm vs. 58.0 ± 9.76 mm, *p* = 0.0055; VD: 76.3 ± 8.55 mm vs. 55.5 ± 10.15 mm, *p* = 0.0079) than females. However, thoracic depth‐to‐width ratios did not differ significantly between male and female greater cane rats on the VD (females: 0.94 ± 0.05; males: 0.94 ± 0.06, *p* = 0.8761) and DV (females: 0.90 ± 0.05; males: 0.91 ± 0.04, *p* = 0.7409) projections. Females had a significantly shorter thoracic inlet diameter than males greater cane rats (16.75 ± 2.63 mm vs. 23.67 ± 2.34 mm, *p* = 0.0024). Measurements of the thoracic inlet diameter, thoracic depth, thoracic width and their ratios are summarised in Table 1.

**TABLE 1:** Radiographic measurements of the thoracic inlet diameter, third rib width, thoracic depth and thoracic width in greater cane rats (*Thryonomys swinderianus*).

Variables	Radiographic projections	Number	Mean ± SD	Range
Thoracic depth (mm)	Right lateral	10	64.00 ± 13.16	43.0–83.0

Thoracic width (mm)	Dorsoventral	10	70.90 ± 13.88	50.0–95.0
	Ventrodorsal	10	68.00 ± 13.81	48.0–93.0

Thoracic depth: thoracic width^∗^		10	0.90 ± 0.04	0.85–0.96

Thoracic depth: thoracic width^∗∗^		10	0.94 ± 0.05	0.87–1.03

Thoracic inlet diameter (mm)	Right lateral	10	21.00 ± 4.27	14.0–27.0

Third rib width (mm)	Right lateral	10	3.60 ± 0.70	3.0–5.0

^∗^Thoracic width measurement taken on the dorsoventral projection.

^∗∗^Thoracic width measurement taken on the ventrodorsal projection.

### 3.1. Musculoskeletal System

All greater cane rats had 13 thoracic vertebrae (Figure 4). The anticlinal vertebra was the 11th thoracic vertebra in all cane rats (Figure 4a). The thoracic spine was almost straight, sloping from caudal to cranial, except caudal to the 11th thoracic vertebra, where it appeared convex dorsally (Figure 4a). The thoracic vertebrae cranial to the anticlinal vertebra had long and slender spinous processes inclined caudally, except the first thoracic vertebra, which had a short spinous process pointing vertically (Figure 4a). Caudal to the anticlinal vertebra, the spinous processes were relatively short, wide and inclined cranially. Generally, vertebral bodies of thoracic vertebrae were more or less of uniform height, with their length increasing as you moved caudally. They appeared rectangular and slender, with the first thoracic vertebra being the shortest and the 13th thoracic vertebra being the longest (Figure 4a). The mamillary processes of 12th and 13th thoracic vertebrae were prominent and projected into the cranial aspect of the respective spinous processes (Figure 4a). The length and height measurements of thoracic vertebrae are presented in Table 2. Most cane rats (9/10) had 6 lumbar vertebrae (Figure 5), while one had five. Lumbar spinous processes were directed cranially and increased in length from cranial to caudal. They appeared relatively wide in a craniocaudal direction (Figure 5a). The mamillary processes were prominently superimposed on spinous processes (Figure 5a). The lumbar vertebrae increased in length from the first to the fifth. The first and fifth lumbar vertebrae were the shortest and longest, respectively. Similarly, the height of the lumbar vertebrae increased from cranial to caudal. The mean length of thoracic and lumbar vertebrae was significantly (*p* < 0.05) smaller in female compared to male greater cane rats. In all greater cane rats, four fused sacral vertebrae were observed. The height and length of lumbar vertebrae are summarised in Table 3.

**TABLE 2:** Radiographic measurements (mm) of height and length of thoracic vertebrae in greater cane rats (*Thryonomys swinderianus*).

Vertebra	Variable	Number	Mean ± SD	Range
T1	Length	10	7.10 ± 1.45	5.00–9.00
	Height	10	4.90 ± 0.88	4.00–6.00

T2	Length	10	7.80 ± 1.32	6.00–10.00
	Height	10	4.90 ± 0.99	4.00–6.00

T3	Length	10	8.20 ± 1.62	6.00–11.00
	Height	10	5.00 ± 0.94	4.00–6.00

T4	Length	10	8.30 ± 1.64	6.00–11.00
	Height	10	5.40 ± 1.17	4.00–7.00

T5	Length	10	8.40 ± 1.65	6.00–11.00
	Height	10	5.40 ± 1.08	4.00–7.00

T6	Length	10	8.70 ± 1.77	6.00–12.00
	Height	10	5.50 ± 1.08	4.00–7.00

T7	Length	10	9.10 ± 1.79	7.00–13.00
	Height	10	5.50 ± 1.08	4.00–7.00

T8	Length	10	9.50 ± 1.90	7.00–13.00
	Height	10	5.50 ± 1.18	4.00–7.00

T9	Length	10	9.90 ± 2.08	7.00–14.00
	Height	10	5.40 ± 0.97	4.00–7.00

T10	Length	10	10.70 ± 2.11	8.00–15.00
	Height	10	5.60 ± 0.97	4.00–7.00

T11	Length	10	11.50 ± 1.90	9.00–15.00
	Height	10	5.10 ± 0.99	4.00–7.00

T12	Length	10	12.40 ± 2.22	9.00–16.00
	Height	10	5.50 ± 0.85	4.00–7.00

T13	Length	10	13.20 ± 2.15	10.00–16.00
	Height	10	5.60 ± 0.97	4.00–7.00

**TABLE 3:** Radiographic measurements (mm) of height and length of lumbar vertebrae in greater cane rats (*Thryonomys swinderianus*).

Vertebra	Variable	Number	Mean ± SD	Range
L1	Length	10	14.10 ± 2.42	10.00–18.00
	Height	10	6.30 ± 1.25	4.00–8.00

L2	Length	10	15.30 ± 2.54	11.00–19.00
	Height	10	6.70 ± 1.16	5.00–8.00

L3	Length	10	16.60 ± 2.72	12.00–21.00
	Height	10	6.90 ± 1.29	5.00–9.00

L4	Length	10	18.10 ± 3.00	13.00–22.00
	Height	10	7.20 ± 1.75	5.00–10.00

L5	Length	10	18.50 ± 2.92	14.00–22.00
	Height	10	7.20 ± 1.48	5.00–10.00

L6	Length	9	17.44 ± 3.17	13.00–22.00
	Height	9	7.44 ± 1.81	5.00–10.00

The sternum was nearly straight, sloping from cranial to caudal. It comprised six sternebrae, including the manubrium sterni and xiphoid process in all cane rats (Figure 4a). The longest sternebra, the manubrium sterni, was tapered cranially, forming a rounded cranial extremity (Figure 4a). The second, third, fourth and fifth sternebrae were slender and rectangular‐shaped. The xiphoid process was the thinnest and a more slender sternebra, elongated craniocaudally. The fifth sternebra was the shortest. Male cane rats had a significant (*p* < 0.05) higher mean length of sternebrae than female cane rats. The length and height measurements of sternebrae are shown in Table 4.

**TABLE 4:** Radiographic measurements (mm) of height and length of sternebrae in greater cane rats (*Thryonomys swinderianus*).

Sternebrae	Variable	Number	Mean ± SD	Range
MS	Length	10	29.30 ± 6.45	19.00–40.00
	Height	10	4.90 ± 1.73	3.00–9.00

ST1	Length	10	10.50 ± 2.46	7.00–14.00
	Height	10	4.50 ± 1.65	3.00–8.00

ST2	Length	10	11.00 ± 2.67	7.00–14.00
	Height	10	4.20 ± 1.14	3.00–6.00

ST3	Length	10	8.90 ± 2.47	5.00–13.00
	Height	10	4.60 ± 0.97	3.00–6.00

ST4	Length	10	8.50 ± 2.68	4.00–12.00
	Height	10	4.50 ± 0.97	3.00–6.00

XP	Length	10	14.30 ± 5.90	8.00–28.00
	Height	10	2.40 ± 0.52	2.00–3.00

All greater cane rats had 13 pairs of ribs, with the last two pairs floating. The slender ribs appeared almost straight, sloping from cranial to caudal except at the costal cartilages (Figure 4a). The latter appeared concave and convex cranially and caudally, respectively. The body of the rib increased in width distally and appeared flared towards the costochondral junction. The former was seen relatively wider in the cranial ribs than the caudal ribs (Figure 4a). The mean width of the third rib was 3.60 ± 0.70 mm (range: 3.00–5.00 mm) (Table 1), and males had a significantly higher mean third rib width than female cane rats (4.00 ± 0.63 mm vs. 3.00 ± 0.00 mm, *p* = 0.0147).

### 3.2. Respiratory System

In all greater cane rats, the diaphragm was seen as a single dome on the VD and DV projections (Figures 2b and 4b). The cranial border of the diaphragmatic cupula was situated at the level of the ninth thoracic vertebra in the majority (9/10) of cane rats on the DV projection. In only one cane rat it was located at the level of the 10th thoracic vertebra. For the VD projection, the cranial border of the diaphragmatic cupula was at the level of the eighth thoracic vertebra in one cane rat. In the remaining cane rats (9/10), it was at the level of the ninth thoracic vertebra. The mean location of the cranial border of the diaphragmatic cupula with respect to thoracic vertebrae did not differ significantly on the VD and DV projections (VD: 8.90 ± 0.32; DV: 9.10 ± 0.32, *p* = 0.1679). In a greater proportion of cane rats (6/10), the diaphragmatic crura were seen superimposed (Figure 2a), whereas in 1/10 and 3/10 cane rats, the crura appeared *Y*‐shaped and parallel, respectively, on the RL projection. On the LL projection the crura frequently (7/10) appeared *Y*‐shaped (Figure 4a) and rarely (3/10) superimposed.

The trachea in the majority (8/10) of the cane rats was observed without mineralised cartilage, while 2 males had it (Figures 2a and 4a). Female cane rats had a significantly lower mean tracheal diameter than male cane rats (5.00 ± 1.15 mm vs. 8.00 ± 0.00 mm, *p* = 0.0002). However, male and female cane rats did not have a significant difference in the mean ratio of the tracheal diameter‐to‐third rib width (male: 2.05 ± 0.35; female: 1.67 ± 0.38, *p* = 0.1429) and tracheal diameter‐to‐thoracic inlet diameter (male: 0.34 ± 0.03; female: 0.30 ± 0.03, *p* = 0.0552). The carina was not clearly visible in all cane rats, and the intersection point of the mainstem bronchi was used to spot its location (Figures 2a and 4a). The former was positioned at the level of the fourth thoracic vertebra on the lateral, DV and VD projections (Figures 2 and 4). With respect to intercostal spaces, the carina was found at the level of the third intercostal space on the lateral projections (Figures 2a and 4a). The mean bifurcation angle of the left and right mainstem bronchi did not differ significantly (*p* = 0.1270) on the DV (47.80 ± 3.77°) and VD (46.40 ± 2.99°) projections. Similarly, gender‐wise, no significant difference in the mean bifurcation angle of the left and right mainstem bronchi was encountered on the DV (male: 47.83 ± 4.31°; female: 47.75 ± 3.40°; *p* = 0.9750) and VD (male: 46.33 ± 3.56°; female: 46.50 ± 2.38°; *p* = 0.9371) projections.

The dorsocaudal tip of the caudodorsal lung fields was seen at the level of the 11th thoracic vertebra in 9/10 and 7/10 cane rats on the RL and LL projections, respectively (Figures 2a and 4a). In one and three cane rats, it was located at the level of the 12th thoracic vertebra on the RL and LL projections, respectively. The position of the dorsocaudal tip of the caudodorsal lung fields did not differ significantly (*p* = 0.3434) on lateral projections. The cranial extension of the cranioventral lung fields on the LL projection was commonly at the first intercostal space (8/10) and infrequently at the first rib (2/10) (Figure 4a). Similarly, on the RL projection, the extension of the cranioventral lung fields was at the first intercostal space in most greater cane rats (7/10). In few cane rats (3/10), the cranial extension was at the first rib. On the VD and DV projections, the cranial end of the right lung was seen wider and extended more cranially than that of the left lung (Figures 2b and 4b). For the DV projection, the cranial end of the right lung was mainly situated at the level of the third rib (6/10) and infrequently at the third intercostal space (1/10) or fourth rib (3/10). The left lung’s cranial end was seen at the level of the fourth rib in 5/10 cane rats. In two and three cane rats, it was positioned at the fourth and third intercostal spaces, respectively. For the VD projection, the cranial end of the right lung was visualised at the level of the third rib in a greater proportion of cane rats (8/10) and infrequently at the level of the second rib (1/10) or third intercostal space (1/10). The left lung’s cranial end was either seen at the third intercostal space (4/10) or the fourth rib (5/10) and hardly at the third rib (1/10). Measurements of organs and structures of the respiratory system in cane rats are indicated in Table 5.

**TABLE 5:** Radiographic findings and measurements of thoracic and abdominal structures/organs in greater cane rats (*Thryonomys swinderianus*).

Variables	Radiographic projections	Number	Mean ± SD	Range
Tracheal diameter (mm)	Right lateral	10	6.80 ± 1.69	4.00–8.00

Tracheal diameter: thoracic inlet diameter	Right lateral	10	0.32 ± 0.03	0.27–0.36

Tracheal diameter: third rib width	Right lateral	10	1.89 ± 0.39	1.33–2.67

Angle between the mainstem bronchi (°)	Dorsoventral	10	47.80 ± 3.77	40.00–53.00
	Ventrodorsal	10	46.40 ± 2.99	40.00–50.00

Location of the dorsocaudal tip of the caudodorsal lung fields with respect to thoracic vertebrae	Right lateral	10	11.10 ± 0.32	11.00–12.00
	Left lateral	10	11.30 ± 0.48	11.00–12.00

Cranial position of the diaphragmatic cupula with respect to thoracic vertebrae	Dorsoventral	10	9.10 ± 0.32	9.00–10.00
	Ventrodorsal	10	8.90 ± 0.32	8.00–9.00

Cardiac silhouette long axis (mm)	Right lateral	10	54.50 ± 10.37	37.00–70.00
	Left lateral	10	53.90 ± 9.68	38.00–68.00
	Dorsoventral	10	55.00 ± 9.71	41.00–68.00
	Ventrodorsal	10	55.00 ± 9.67	40.00–70.00

Cardiac silhouette short axis (mm)	Right lateral	10	36.90 ± 8.33	26.00–53.00
	Left lateral	10	39.10 ± 7.67	28.00–54.00
	Dorsoventral	10	44.50 ± 9.28	33.00–64.00
	Ventrodorsal	10	43.60 ± 9.65	31.00–64.00

Vertebral heart size	Right lateral	10	9.70 ± 0.33	9.20–10.20
	Left lateral	10	9.83 ± 0.27	9.40–10.30
	Dorsoventral	10	10.46 ± 0.51	9.60–11.20
	Ventrodorsal	10	10.45 ± 0.39	9.90–11.00

Cardiodiaphragmatic contact (mm)	Right lateral	10	16.60 ± 6.54	0.00–22.00
	Left lateral	10	17.40 ± 7.15	0.00–27.00
	Dorsoventral	10	19.20 ± 2.86	16.00–26.00
	Ventrodorsal	10	14.70 ± 5.89	0.00–22.00

Cardiothoracic ratio	Dorsoventral	10	0.63 ± 0.05	0.56–0.69
	Ventrodorsal	10	0.64 ± 0.04	0.57–0.69

Cardiac silhouette size with respect to intercostal spaces	Right lateral	10	2.95 ± 0.28	2.50–3.50
	Left lateral	10	3.00 ± 0.33	2.50–3.50

CVC diameter (mm)	Right lateral	10	7.20 ± 1.93	4.00–11.00

CVC diameter: vertebral length above carina	Right lateral	10	0.86 ± 0.12	0.67–1.00

Angle of gastric axis (°)	Right lateral	10	77.60 ± 5.70	70.00–92.00

Small intestinal diameter (mm)	Right lateral	10	3.80 ± 0.42	3.00–4.00

Small intestinal diameter: height of the second lumbar vertebra	Right lateral	10	0.58 ± 0.06	0.50–0.67

### 3.3. Cardiovascular System

On lateral radiographic projections, the CS was seen as ovoid in all greater cane rats (Figures 2a and 4a). The poorly seen cranial border of the CS was situated mainly at the level of the second rib (9/10) and rarely at the first intercostal space (1/10) on the lateral projections (Figures 2a and 4a). On the RL projection, the caudal border of the CS was frequently seen at the level of the fifth rib (8/10) and hardly at the fourth intercostal space (2/10) (Figure 2a). Similarly, on the LL projection it was positioned at the level of the fifth rib in a greater proportion (7/10) of cane rats (Figure 4a). In the remaining three cane rats, it was seen at the fourth (2/10) and fifth (1/10) intercostal spaces. In most cane rats, the CS size was 3.0 intercostal spaces on the RL (7/10) and LL (6/10) projections. Rarely, the CS occupied 2.5 (RL: 2/10; LL: 2/10) or 3.5 (RL: 1/10; LL: 2/10) intercostal spaces. The mean CS size with respect to intercostal spaces obtained from the RL projection (2.95 ± 0.28) did not differ significantly (*p* = 0.3434) from the mean value obtained from the LL projection (3.00 ± 0.33). In 9/10 cane rats cardiodiaphragmatic contact was present (Figures 2a and 4a). The extent of cardiodiaphragmatic contact did not differ significantly between the two lateral projections (RL: 16.60 ± 6.54 mm; LL: 17.40 ± 7.15 mm; *p* = 0.4945). Additionally, the mean cardiodiaphragmatic contact did not vary significantly between male and female cane rats on the lateral projections (LL: *p* = 0.7030; RL: *p* = 0.6762).

The CS was seen as ovoid in all cane rats on the VD and DV projections (Figures 2b and 4b). On the VD projection cardiodiaphragmatic contact was found in 9/10 cane rats, whereas on the DV projection, it was present in all rats. Cane rats had a significant extensive cardiodiaphragmatic contact on the DV projection than on the VD projection (DV: 19.20 ± 2.86 mm; VD: 14.70 ± 5.89 mm; *p* = 0.0097). The extent of cardiodiaphragmatic contact did not vary significantly between male and female cane rats on the VD (*p* = 0.9043) and DV (*p* = 0.3052) projections. The CS apex on the VD projection was at the midline and slightly to the left haemithorax in 4/10 and 6/10 cane rats, respectively (Figure 4b). On the DV projection, the CS apex was mainly seen slightly to the left haemithorax (8/10) and occasionally at the midline (2/10) (Figure 2b).

Male cane rats had a significant (*p* < 0.05) higher mean CS long axis and short axis measurements on the lateral, DV and VD projections than female cane rats. However, the VHS did not vary significantly between male and female cane rats on the LL (female: 9.88 ± 0.15; male: 9.80 ± 0.34; *p* = 0.6937), RL (female: 9.73 ± 0.17; male: 9.68 ± 0.42; *p* = 0.8565), DV (female: 10.40 ± 0.73; male: 10.50 ± 0.37; *p* = 0.7791) and VD (female: 10.43 ± 0.46; male: 10.47 ± 0.38; *p* = 0.8786) projections. Additionally, no significant difference was encountered on the VHS from the LL versus RL (*p* = 0.1803) and VD versus DV (*p* = 0.9372). The VHS calculated from the lateral projections differs significantly (*p* < 0.05) from the VHS calculated from the VD and DV projections. Cardiothoracic ratios calculated on the VD and DV projections did not differ significantly (*p* = 0.0747). Further, no significant difference was observed on the cardiothoracic ratio between male and female cane rats on the DV (female: 0.63 ± 0.05; male: 0.63 ± 0.05; *p* = 0.9208) and VD (female: 0.64 ± 0.03; male: 0.65 ± 0.05; *p* = 0.8521) projections.

On the lateral projections the descending aorta was not clearly seen in all greater cane rats (Figures 2a and 4a). On the DV and VD projections the lateral margin of the descending aorta was clearly seen in 9/10 and 8/10 cane rats, respectively (Figures 2b and 4b). The CVC was clearly visible on the lateral projections in all cane rats (Figures 2a and 4a). Male cane rats had significantly higher mean CVC diameter than female cane rats (male: 8.17 ± 1.60 mm; female: 5.75 ± 1.50 mm; *p* = 0.0437). The mean ratio of the CVC diameter‐to‐length of the thoracic vertebra above the carina was almost similar in male and female cane rats (male: 0.87 ± 0.12; female: 0.84 ± 0.14; *p* = 0.7160). Measurements of organs and structures of the cardiovascular system are provided in Table 5.

### 3.4. Digestive System

The mean angle of the gastric axis was 77.6 ± 5.7° and did not vary significantly between male and female greater cane rats (male: 79.17 ± 6.43°; female: 75.25 ± 3.86°; *p* = 0.3108). The small intestines could be distinguished from the large intestine (Figure 5). The thickness of the small intestine did not differ significantly in male and female cane rats (female: 3.50 ± 0.58 mm; male: 4.00 ± 0.00 mm; *p* = 0.0598). However, female cane rats had a significantly higher mean ratio of the small intestinal thickness‐to‐height of the second lumbar vertebra (female: 0.64 ± 0.04; male: 0.54 ± 0.04; *p* = 0.0042). The large intestinal thickness was not measured due to its high variability in shape and size. Measurements of the gastric axis angle and thickness of the small intestine with its ratio to the height of the second lumbar vertebra are presented in Table 5.

### 3.5. Urinary System

Kidneys and ureters were not visualised on abdominal radiographs in all greater cane rats. The urinary bladder was poorly seen in 4/10 cane rats and appeared either ovoid (3/4) or oblong (1/4) shaped (Figure 5a).

### 3.6. Other Findings

On the DV and VD projections, the cranial mediastinum was wider than the superimposed thoracic spine in all greater cane rats (Figures 2b and 4b). Grainy materials with mineral opacity were seen in 2/10 greater cane rats in the cranial abdomen (Figure 4b).

## 4. Discussion

Radiography is an important and most frequently used diagnostic imaging modality in veterinary clinical practices due to its relative availability, simple interpretation, rapidity, non‐invasiveness and lower cost [19]. Normal radiological anatomy of a particular animal species provides the basis for radiographic interpretation and diagnosis [23]. Moreover, comparison of radiographic measurements of the thoracic and abdominal structures/organs to relevant skeletal structures provides their relative size for objective assessment and reduces the variability due to different body sizes within an individual species [49, 53]. This report has provided the normal radiological reference values and ranges for thoracic and abdominal organs/structures. The shape, location, number, opacity and margination of thoracic and abdominal structures/organs in greater cane rats are described. Therefore, it is the first comprehensive thoraco‐abdominal radiographic reference for greater cane rats and provides additional radiographic information to recent publications on the greater cane rat [40–42]. This information is valuable in biomedical and related research, veterinary clinical practices, and in teaching comparative veterinary anatomy.

The mean thoracic depth‐to‐width ratios obtained on the DV (0.90) and VD (0.94) projections in this study categorise the greater cane rat as having an intermediate (normal) thoracic conformation [49], similarly to the southern giant pouched rat [23]. Nevertheless, the thoracic depth‐to‐width ratios differed significantly on the DV and VD projections. The sloping pattern of the thoracic spine, that is, from caudal to cranial in cane rats, is analogous to southern giant pouched rats [23], and most likely it is an adaptive feature for surviving under reeds, holes and crevices along riverbeds.

Thoracic and abdominal skeletal structures show variations in morphology, number and size within and between species [54]. In this study all cane rats had 13 thoracic vertebrae with corresponding rib pairs like other reported rodents like mole rats [55], southern giant pouched rats [23], Norway albino rats [56], common capybaras [57] and black‐rumped agouti [25]. In a study which involved guinea pigs [58], it was reported that a greater proportion (75%) had 13 thoracic vertebrae which is similar to cane rats. Though the greater cane rat has shown a greater uniformity in the number of thoracic vertebrae, that is, 13, their morphology and size vary from cranial to caudal. The common observation of the 11th thoracic vertebra as an anticlinal vertebra in this species contrasts with the findings in the common capybara [57] and southern giant pouched rat [23]. The 10th and 12th thoracic vertebrae were reported as anticlinal vertebrae in the southern giant pouched rat [23] and common capybara [57], respectively.

The common (9/10) observation of six lumbar vertebrae in cane rats is analogous to reported studies in the guinea pig [58], Norway albino rat [56], mole rat [55], common capybara [57] and giant rat [59]. Anatomical studies of the spinal cord which involved 12 greater cane rats [60] reported the presence of five lumbar vertebrae, which is contrary to findings of this study. A discrepancy in the number of lumbar vertebrae, that is, 5–6, which has been encountered in this study in the cane rats, has also been documented in guinea pigs [58]. The prominent mamillary processes seen in the caudal thoracic and lumbar vertebrae should not be misinterpreted as a congenital abnormality. The mamillary processes can be distinguished radiographically from spinous processes by being relatively short and narrow craniocaudally compared to their respective spinous processes. The occurrence of four fused sacral vertebrae in cane rats is comparable to a previously reported study in cane rats by Mustapha et al. [60] and other rodents [55, 57, 58].

In this study, all cane rats had a total of six sternebrae, including the xiphoid process and manubrium sterni. This finding is like that of southern giant pouched rats [23] and Norway albino rats [56]. Contrarily, the reported total number of sternebrae, including the xiphoid process and manubrium sterni, in other species of rodents like the mole rat [55], common capybara [57] and black‐rumped agouti [25] is seven. The characteristic bullet‐shaped manubrium sterni is different from a reported study in the southern giant pouched rat [23].

Information on the normal radiological anatomy of the thoracic and abdominal skeletal structures is important for detecting various diseases and conditions such as those associated with a change in opacity, position, shape and number of skeletal structures. Moreover, it is useful for species identification [61]. Additionally, skeletal structures of the thorax and abdomen are utilised as landmarks in diagnostic imaging, surgical procedures, and clinical and physical examinations.

In other species of rodents [15, 17, 23, 26, 62], the poor visibility of the cranial border of the CS on the lateral projections has been explained as the result of the superimposition of the cranioventral mediastinal tissues with the cranial part of the CS, which might also explain the cane rat. The poorly seen cranial border of the CS may pose a challenge in the objective evaluation of the CS by VHS on the lateral projections. However, in such scenarios, the DV or VD projection may be used in this species since the CS margins are clearly seen. In rodents various efforts to enhance visibility of the heart and CS margins on thoracic radiographs have been made using contrast media, manual ventilation and positive pressure breath hold during exposure [15, 17, 26, 62]. The location of the CS apex, either to the midline or slightly to the left haemithorax in cane rats, is different from southern giant pouched rats [23]. In southern giant pouched rats, the apex of the CS is positioned relatively far away from the midline [23].

The CS size with respect to intercostal spaces obtained in this study provides a quick reference for subjective evaluation of the CS size on the lateral projections. Additionally, the lack of a statistically significant difference in the CS width with respect to intercostal spaces obtained on the RL (2.95) versus LL (3.00) projections indicates that the LL projection may also be used for subjective evaluation of the CS size in cane rats. However, the subjective evaluation of the CS size with respect to intercostal spaces is to be affected by respiratory phase, variations in the axis of the heart, imprecise measurement points and superimposition of the ribs [49].

The VHS is an objective method which is used for evaluation of the CS size on radiographic [50] and computed tomographic (CT) [27] images of the thorax. It compares the long and short axis measurements of the CS to thoracic vertebrae [49] for the detection of changes in the CS size. The mean VHS obtained in this study in cane rats on the lateral projections (RL: 9.70; LL: 9.83) is higher than the reported mean values in Wistar rats (RL: 7.22, 7.80, and 7.97; LL: 7.34) [17, 62, 63], Sprague–Dawley rats (RL: 7.70) [15], guinea pigs (RL: 7.10 and 7.70; LL: 6.9) [64, 65], chinchillas (RL: 8.94; LL: 8.89) [27], black‐tailed prairie dogs (RL: 7.12; LL: 7.15) [26], laboratory mice (RL: 9.10; LL: 9.40) [66], black‐rumped agoutis (RL: 8.04; LL: 8.12) [25], and southern giant pouched rats (RL: 9.49) [23]. Additionally, the mean VHS values in this species on the DV (10.46) and VD (10.45) projections are larger than the mean values in Wistar rats (DV: 7.90) [17], guinea pigs (VD: 9.2) [65], and Sprague–Dawley rats (DV: 7.9; VD: 7.5) [15]. Interestingly, the laboratory mice [66] and southern giant pouched rats [23] have been documented to have higher mean VHS on the DV (laboratory mice: 12.60; southern giant pouched rats: 10.59) and VD (laboratory mice: 12.20) projections compared to cane rats. The higher mean VHS value in southern giant pouched rats compared to cane rats on the DV projection might be due to the elongated appearance and oblong‐shaped CS in southern giant pouched rats [23]. This might also explain the laboratory mice. A previous study [40] reported lower mean VHS values on the lateral projections in adult cane rats compared to the observations of this study. The cause of this variation cannot be ascertained clearly.

In the present study the mean VHS values obtained from the two lateral projections did not differ significantly, which justifies the use of either the RL or LL projection for objective evaluation of the CS in cane rats [50]. Similar results have been documented in other rodents [25, 27, 63, 64, 66]. The lack of a statistically significant difference between the mean VHS values on the DV versus VD projections is analogous to the reported study in laboratory mice [66] and further supports the use of either the DV or VD projection for evaluation of the CS size using a VHS system in cane rats. Contrarily, in Sprague–Dawley rats [15] the VHS values on the DV projection were larger than those obtained from the VD projection. In greater cane rats, the mean VHS values calculated on the DV and VD projections were significantly higher than those calculated on the lateral projections. This is because on the DV and VD projections there is magnification of the CS as a result of increased distance between the cassette and the heart [49]. Additionally, on the lateral projections the long‐axis measurement includes only the left atrium and ventricle, whereas on the VD and DV projections the long‐axis measurement extends from the right atrium to the left ventricle [49]. Higher VHS values on the DV/VD projections compared to lateral projections are similar to studies in southern giant pouched rats [23], laboratory mice [66] and guinea pigs [65]. In the present study male cane rats had significantly higher long‐axis and short‐axis measurements compared to females. However, when these measurements were compared to the length of thoracic vertebrae, no significant difference was encountered between the two genders. This highlights the importance of using CS size to relevant skeletal structure ratios as reference values. Female greater cane rats have been documented to be smaller than male greater cane rats [67].

The cardiothoracic ratio has limitations for the evaluation of the CS size in the presence of different thoracic conformations within a species [49]. The cardiothoracic ratios in cane rats (DV: 0.63; VD: 0.64) in this study are larger than the reported ratios in black‐tailed prairie dogs (0.48) [26] and southern giant pouched rats (0.50) [23]. However, they were smaller than the reported ratio in Wistar rats (0.89) [63]. Variations in cardiothoracic ratios among different species of mammals are most likely due to different thoracic conformations.

Various congenital and acquired cardiac and extracardiac diseases and abnormalities such as pericardial effusions, dilated cardiomyopathy, hypertrophic cardiomyopathy, vertebral abnormalities, pectus excavatum, hypovolaemia, dextrocardia, thoracic masses, mediastinal shift, right atrial enlargement, left atrial and ventricular enlargement can alter the CS size, shape and position [68]. Knowledge of the normal CS shape, position and size in a species is essential for identifying abnormalities. Among the three methods which have been used in this study for evaluation of the CS size in cane rats, the VHS system is preferred due to a good correlation that exists between the body length and heart weight [49]. Therefore, it is valuable for initial assessment of the CS size and for monitoring the progression of the CS enlargement over time in individuals [49].

Cardiovascular diseases lead to changes in the size of associated major blood vessels supplying and returning blood from the peripheral circulation. Right‐sided congestive heart diseases such as dilated cardiomyopathy, pulmonic stenosis and pericardial disease lead to dilatation of the CVC [69]. The ratio of the CVC diameter‐to‐length of the thoracic vertebra above the carina in cane rats (0.86) is almost similar to southern giant pouched rats (0.87) [23] and useful in the recognition of changes in the CVC diameter. The CVC diameter varies with body size [69]. The objective method for assessing major blood vessels diameter, including the CVC is by comparing their size with relevant skeletal structures [69]. This overcomes the size variability within a species.

Determination of the normal diameter of the trachea in individual species is of paramount importance in recognition of diseases and conditions that change the luminal diameter of the trachea, like tracheal hypoplasia, collapse and respiratory difficulty [46, 68]. The tracheal dimeter‐to‐thoracic inlet diameter ratio in cane rats (0.32) is nearly comparable to a reported value in the southern giant pouched rat (0.27) [23]. The poor visibility of the carina in cane rats has also been documented in the southern giant pouched rat [23]. The position of the carina in cane rats with respect to intercostal spaces and thoracic vertebrae observed in this study, that is, the third intercostal space and fourth thoracic vertebra, respectively, is analogous to other rats [15, 17, 23, 62]. The carina may be placed caudal to its normal location because of cranial mediastinal masses and pleural effusions [70]. An increase in tracheal bifurcation angle is associated with left atrial enlargement, large caudal oesophageal masses and hilar lymphadenopathy [68]. In cane rats, the tracheal bifurcation angle can either be measured on the DV or VD projection due to the lack of a significant difference in the measured tracheal bifurcation angle between the two projections. In this study all cane rats had a wider cranial mediastinum than the superimposed spine, which is comparable to southern giant pouched rats [23]. This is a normal finding in cane rats and should not be misinterpreted as a widening as a result of a craniomediastinal mass.

Generally, the abdominal serosal detail was poor in all cane rats. Wild cane rats have a smaller amount of fat than captive cane rats, which contributes to reduced abdominal serosal detail. The wild cane rats are more active than captive cane rats, and their diet is higher in fibre and lower in fats. The presence of fat in the abdomen provides contrast for differentiation of abdominal organs. A greater cane rat is a monogastric herbivore with a well‐developed caecum as a hindgut‐fermenting organ [71]. The caecum occupies up to 66.70% of the left upper abdominal cavity [71] and is usually filled with gas, reducing abdominal detail on the RL projection. Abdominal organ(s) may not be seen because of being obscured by gas. Moreover, the thick and coarse nature of the cane rat’s hair coat may have provided additional density, which contributed to poor detail of the abdomen.

Measurement of the angle of the gastric axis on abdominal radiographs is useful in the determination of changes in the liver size [68]. The gastric axis may be shifted cranially in the presence of diaphragmatic rupture/hernia, hiatal hernia, gastro‐oesophageal intussusception, peritoneopericardial diaphragmatic hernia and pathological conditions that reduce liver size like hepatic cirrhosis and portosystemic shunts [68]. Expansion of the thorax and an enlarged liver result in caudal displacement of the gastric axis [68]. The ratio of the small intestinal thickness‐to‐height of the second lumbar vertebra in cane rats will assist in recognition of obstructive diseases/conditions of the small intestine. The presence of grainy materials with mineral opacity observed in the cranioventral abdomen in two cane rats in this study is believed to be a combination of sand and gravel grains. A similar finding was reported in southern giant pouched rats [23].

Further studies on diagnostic imaging are recommended in this species, such as contrast radiography, ultrasonography and CT. The CT will provide a cross‐section anatomy of the thorax and abdomen without the superimposition of thoracic and abdominal organs/structures. Hence, it will allow visualisation of organs/structures which were not clearly seen on plain radiographic examination, like the aorta, pulmonary vessels, bronchial tree and kidneys. Echocardiography will complement thoracic radiography by providing reference ranges for chambers of the heart and wall measurements since a normal CS on thoracic radiographs does not always rule out the presence of a cardiovascular disease. Abdominal ultrasonographic and contrast radiographic studies are also recommended in cane rats to allow evaluation of abdominal organs such as the urinary bladder, kidneys, spleen and adrenal glands which were not clearly visible or not visible at all on plain abdominal radiographs.

## 5. Conclusions

This study has described the normal radiographic appearance of the thorax and abdomen in greater cane rats. Measurements of sternebrae, thoracic and lumbar vertebrae are provided. Additionally, it has provided reference ranges, such as the angle between the mainstem bronchi, tracheal diameter and gastric axis angle. Moreover, different ratios like cardiothoracic, VHS, tracheal diameter to thoracic inlet diameter and CVC diameter to length of thoracic vertebra above the carina in greater cane rats have been calculated. Data generated from this study will provide reference for further biomedical studies and for teaching comparative veterinary anatomy for undergraduate and postgraduate students. Further, information from this study will increase awareness and use of diagnostic radiography and other imaging modalities in cane rats for diagnosis and monitoring healing progress of various pathological diseases/conditions.

## 6. Limitations of the Study

Authors were not blinded to sex and body weight of greater cane rats. Additionally, the heart function of cane rats was not evaluated by other methods such as through electrocardiography and echocardiography. Lastly, the intra‐observer variability was not assessed.

## Author Contributions

Mazengo Masigati: data curation, formal analysis, funding acquisition, investigation, methodology, project administration, visualization, writing–original draft and writing–review and editing; Mirende Kichuki: supervision and writing–review and editing; Modesta Makungu: conceptualization, supervision and writing–review and editing.

## Funding

This research was funded by the Sokoine University of Agriculture.

## Conflicts of Interest

The authors declare no conflicts of interest.

## Data Availability

The data that support the findings of this study are available from the corresponding author upon reasonable request.
